# Fecal microbiota transplantation from HUC-MSC-treated mice alleviates acute lung injury in mice through anti-inflammation and gut microbiota modulation

**DOI:** 10.3389/fmicb.2023.1243102

**Published:** 2023-09-28

**Authors:** Feng Hua, Enhai Cui, Lu Lv, Bin Wang, Liqin Li, Huadong Lu, Na Chen, Wenyan Chen

**Affiliations:** ^1^Department of Respiratory and Critical Care Medicine, Huzhou Central Hospital, Affiliated Huzhou Hospital, Zhejiang University School of Medicine, Huzhou, China; ^2^Traditional Chinese Medicine Key Laboratory Cultivation Base of Zhejiang Province for the Development and Clinical Transformation of Immunomodulatory Drugs, Huzhou, China

**Keywords:** fecal microbiota transplantation, human umbilical cord mesenchymal stromal cells, acute lung injury, inflammation, gut microbiota

## Abstract

**Introduction:**

Acute lung injury (ALI) is a severe respiratory tract disorder facilitated by dysregulated inflammation, oxidative stress and intestinal ecosystem. Fecal microbiota transplantation (FMT) is a rapid method for gut microbiota (GM) reconstruction. Furthermore, our previous studies have confirmed that human umbilical cord mesenchymal stromal cells (HUC-MSCs) can alleviate ALI by improving GM composition. Therefore, we aimed to explore the efficacy and mechanism of FMT from HUC-MSCs-treated mice on ALI.

**Methods:**

In brief, fresh feces from HUC-MSCs-treated mice were collected for FMT, and the mice were randomly assigned into NC, FMT, LPS, ABX-LPS, and ABX-LPS-FMT groups (*n* = 12/group). Subsequently, the mice were administrated with antibiotic mixtures to deplete GM, and given lipopolysaccharide and FMT to induce ALI and rebuild GM. Next, the therapeutic effect was evaluated by bronchoalveolar lavage fluid (BALF) and histopathology. Immune cells in peripheral blood and apoptosis in lung tissues were measured. Furthermore, oxidative stress- and inflammation-related parameter levels were tested in BALF, serum, lung and ileal tissues. The expressions of apoptosis-associated, TLR4/NF-κB pathway-associated, Nrf2/HO-1 pathway related and tightly linked proteins in the lung and ileal tissues were assessed. Moreover, 16S rRNA was conducted to assess GM composition and distribution.

**Results:**

Our results revealed that FMT obviously improved the pathological damage of lung and ileum, recovered the immune system of peripheral blood, decreased the cell apoptosis of lung, and inhibited inflammation and oxidative stress in BALF, serum, lung and ileum tissues. Moreover, FMT also elevated ZO-1, claudin-1, and occludin protein expressions, activating the Nrf2/HO-1 pathway but hindering the TLR4/NF-κB pathway. Of note, the relative abundances of Bacteroides, *Christensenella, Coprococcus*, and *Roseburia* were decreased, while the relative abundances of *Xenorhabdus, Sutterella*, and *Acinetobacter* were increased in the ABX-LPS-FMT group.

**Conclusion:**

FMT from HUC-MSCs-treated mice may alleviate ALI by inhibiting inflammation and reconstructing GM, additionally, we also found that the TLR4/NF-κB and Nrf2/HO-1 pathways may involve in the improvement of FMT on ALI, which offers novel insights for the functions and mechanisms of FMT from HUC-MSCs-treated mice on ALI.

## 1. Introduction

Acute lung injury (ALI) is a serious disorder characterized by acute, inflammatory, and diffuse damage that occurs in the lungs (Jiang et al., 2021). In the United States, 200,000 patients are diagnosed with ALI, and the mortality rate is as high as 40% (Costa et al., 2017). Despite great efforts, the mortality of ALI remains considerable, and definitive therapies are still lacking (Wang et al., 2021). So far, lung protective ventilation represents the only beneficial strategy for ALI patients, but the extensive adoption of the strategy contributes to the occurrence of ventilator-induced lung injury (Li et al., 2017). Thus, new and efficacious treatment strategies for ALI are desperately required.

Following ALI, numerous inflammatory factors were generated and released, which exacerbate lung injury (Yang et al., 2020). The triggered inflammation not only causes oxidative stress but also results in apoptosis in the lungs, both of which play key roles in ALI initiation and progression (Jia et al., 2021). As is well-known, the TLR4/NF-κB pathway is a classic signal pathway responsible for regulating inflammation (Zhang et al., 2019a). Currently, research has found that inflammation in lipopolysaccharide (LPS)-induced ALI can be alleviated *via* the inhibition of the TLR4/NF-κB pathway (Liu et al., 2020b). In addition, the Nrf2/HO-1 pathway is also a crucial pathway for antioxidant defense (Su et al., 2020). Following oxidative stress, Nrf2 separates from Keap-1, combines with the antioxidant response element (ARE), and then elevates the expression of some antioxidant-encoding proteins, which protect against oxidative stress in ALI (Li Y. et al., 2023). Bi et al. have revealed that activating the Nrf2/HO-1 pathway can reduce oxidative stress in ALI mice. Hence, looking for new drugs that can suppress inflammation and oxidative stress by modulating the TLR4/NF-κB and Nrf2/HO-1 pathways is an efficient approach to treating ALI.

Gut microbiota (GM) interacts with the host and is pivotal for controlling host health (Bi et al., 2020). Recent research has demonstrated that GM can impact the lung function of ALI *via* the “gut–lung axis” (Kapur et al., 2018). Specifically, disordered GM will break down intestinal barrier integrity, so as to activate the immune system, facilitate bacterial displacement, induce migration of bacteria to the lungs, and exacerbate lung inflammation and oxidative stress (Tang et al., 2021). Fecal microbiota transplantation (FMT) is a simple and fast method for gut bacterial transplantation. Following FMT, the composition of GM in the recipient is similar to that in the donor (Le Bastard et al., 2018). Published studies have found that FMT from normal mice can relieve ALI by restoring GM composition, inhibiting inflammation (Li et al., 2020), and reducing oxidative stress (Tang et al., 2021). In addition, our previous study has also revealed that human umbilical cord mesenchymal stromal cells (HUC-MSCs) can attenuate LPS-stimulated ALI by modulating lung–gut microbiota. In addition, we also observed that compared with the sham mice without HUC-MSC treatment, there were three microflora with upregulated abundance and nine microflora with downregulated abundance in the feces of mice treated with HUC-MSCs (Supplementary Figure 1). However, the effect and mechanism of FMT from HUC-MSC-treated mice on ALI remain unknown.

Hence, in this study, we performed animal experiments to explore whether FMT from HUC-MSC-treated mice can alleviate LPS-induced ALI by modulating inflammation and GM composition. These findings may offer a novel effective therapeutic option for ALI.

## 2. Materials and methods

### 2.1. Animals

C57BL/6J mice (6–8 weeks old, 20.0 ± 2 g) were supplied by Beijing Vital River Laboratory Animal Technology Co., Ltd (China, license No. SCXK (Beijing) 2016-0006). The mice were all housed in a specific pathogen-free environment with controlled temperature (22–26°C), humidity (40%−60%), and lighting (12/12-h light–dark cycle). All mice received water and standard food freely. All animal-related experiments were carried out with the evaluation and supervision of the Animal Experimentation Ethics Committee of Zhejiang Eyong Pharmaceutical Research and Development Center [certificate No. SYXK (Zhe) 2021-0033] and in compliance with the guidelines of the Institutional Animal Care and Use Committee.

### 2.2. Animal grouping and FMT treatment

All mice were allowed to acclimate to the conditions of the animal house for 7 days before the experiments. A mouse died during the adaptation phase, and subsequently, the surviving mice were randomized into five groups (*n* = 12/group): Group A: normal control group (NC); Group B: normal mice received FMT from HUC-MSC-treated mice group (FMT, the protocol of FMT is presented below); Group C: LPS-stimulated group (LPS); Group D: mice received antibiotics (ABX) and LPS stimulation group (ABX-LPS); Group E: mice treated with ABX, stimulated by LPS, and then received FMT from the HUC-MSC-treated mice group (ABX-LPS-FMT).

**Figure 1:**
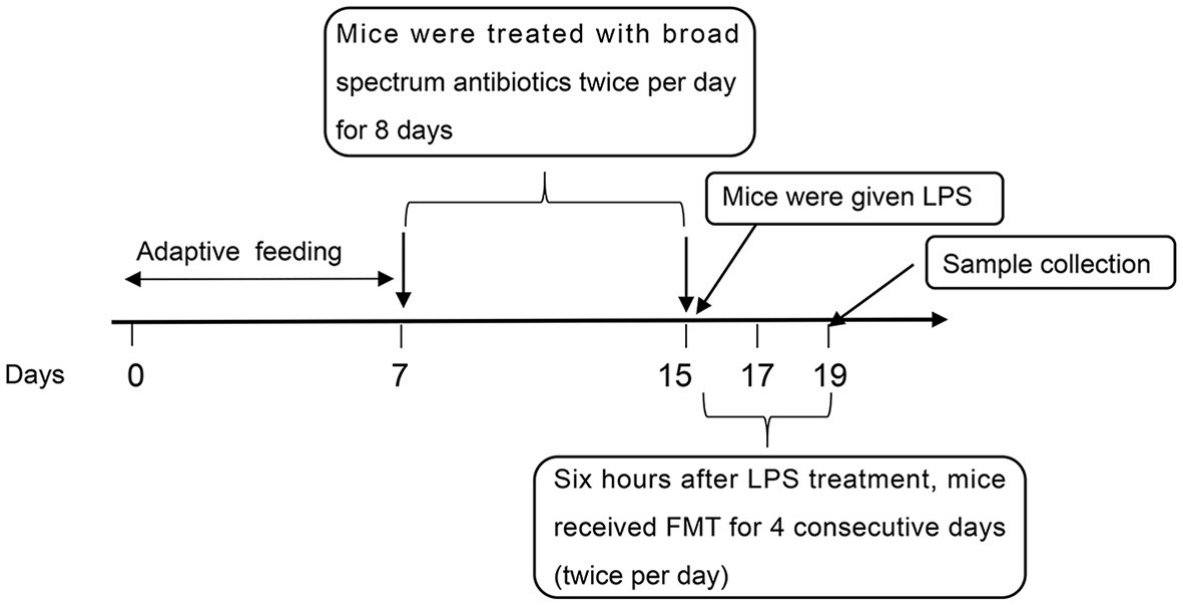
The flow of the experiment. LPS, lipopolysaccharide; FMT, fecal microbiota transplantation.

The flow of the experiment is shown in Figure 1. In brief, mice from the ABX-LPS and ABX-LPS-FMT groups received broad-spectrum antibiotics (0.1 mg/g neomycin, 0.1 mg/g ampicillin, 0.1 mg/g metronidazole, and 0.05 mg/g vancomycin) by gavage twice per day for 8 days; the step was utilized to remove the GM of the mice. The other mice were treated with an equal volume of sterile water intragastrically. Subsequently, the LPS, ABX-LPS, and ABX-LPS-FMT groups were administered with 100 μl LPS (10 mg/kg) intraperitoneally to construct the ALI model, and the remaining mice were perfused with the same amount of sterile saline (Yang et al., 2021). After 6 h, mice in the FMT and ABX-LPS-FMT groups received FMT treatment; fresh fecal samples were collected from normal mice that had received HUC-MSC treatment (in short, 6–8-week-old male C57BL/6 mice were treated with 100 μl 0.9% NaCl). After 6 h, the mice were injected intraperitoneally with 0.5 ml of phosphate-buffered saline (PBS) containing HUC-MSCs (2 × 10^6^ cells/ml, iCell Bioscience). After 3 days of HUC-MSC intervention, the fecal samples of the mice were collected (Wu et al., 2022). Then, the fecal samples were homogenized and diluted to a final concentration of 50 mg/ml using sterile saline. Afterward, the fecal samples were centrifuged, and the supernatant filtered by filters (70 μm) was applied for FMT. FMT was achieved by giving 200 μl fecal sample supernatant to the recipient mice intragastrically for four consecutive days (twice per day) (Wu et al., 2021). Correspondingly, mice in other groups were treated with an equal volume of sterile saline. The ALI model induced by LPS is deemed to be reliable and stable (Yang et al., 2021).

### 2.3. Specimen collection

On the day of the last injection, fecal samples from the NC, FMT, LPS, and ABX-LPS-FMT groups were collected. Then, the mice were euthanized using 5% isoflurane, and the blood from the abdominal aorta was collected and centrifuged to obtain serum. After bronchoalveolar lavage fluid (BALF) was collected from the left lung, the right lung was harvested to evaluate the lung wet to dry (W/D) ratio and other indicators. Additionally, ileal tissues were removed from the mice and kept for further tests.

### 2.4. Collection and analysis of BALF

The trachea cannula of the left lung was washed with 0.7 ml of PBS repeatedly. The collected BALF was centrifuged, and the supernatant was utilized to determine BALF protein concentration and inflammatory cytokines. The cell pellets were resuspended by PBS, and then, the total cell number in BALF was counted by cell counting plate. Furthermore, the number of neutrophils in BALF was calculated by Wright–Giemsa staining.

### 2.5. Lung W/D ratio

The middle lobe of the right lung was excised and weighed immediately to obtain the wet mass of the lungs. Next, these lung tissues were dehydrated for 48 h at 80°C, to obtain the dry mass of the lungs. Finally, the lung W/D ratio was calculated to evaluate pulmonary edema.

### 2.6. Detection of malondialdehyde, glutathione, superoxide dismutase, and myeloperoxidase levels in the lung tissues

Lung tissue samples were rinsed with PBS and then minced into pieces. The tissues were fully homogenized on ice with PBS. Then, the homogenates were centrifuged; the supernatant was obtained to measure the levels of detection of malondialdehyde (MDA), glutathione (GSH), superoxide dismutase (SOD), and myeloperoxidase (MPO) by commercial kits (A003-1; A006-2-1; A001-1; A044-1-1; Nanjing Jiancheng Bioengineering Institute, China).

### 2.7. Enzyme-linked immunosorbent assay (ELISA) analysis

The homogenates of ileal tissues were prepared as lung tissue. Then, commercial enzyme-linked immunosorbent assay (ELISA) kits were applied to test the contents of interleukin-1β (IL-1β, ml063132-1), interleukin-6 (IL-6, ml002293-1), and tumor necrosis factor-a (TNF-a, ml002095-1) in the BALF, serum, lung, and ileal tissues, following the manufacturer's instructions. All ELISA kits were bought from Shanghai Enzyme-linked Biotechnology Co., Ltd (China).

### 2.8. Peripheral blood immune cell analysis

The whole blood of the mice was collected and anticoagulated with heparin sodium. Then, the blood was treated with corresponding antibodies for 0.5 h at room temperature. Specifically, Tc cells were evaluated by anti-CD3e (F2100301), anti-CD8a (F2100810), and anti-CD4 (F2100403) antibodies. DCs were measured using anti-CD11c (F21011C03) and anti-CD86 (F2100810) antibodies. NK cells were examined with anti-CD49b (F21049B03) and anti-CD3e antibodies. Tregs were tested using anti-CD4, anti-CD25 (F2102504), and anti-Foxp3 (F21FP302) antibodies. Finally, the mixture was centrifuged. After removing the supernatant, the pellet was resuspended in PBS and analyzed using flow cytometry. All antibodies used in the experiment were supplied by Liankebio (China).

### 2.9. Histological analysis

The collected lung and ileal tissues were rinsed with PBS, followed by fixation and dehydration using 4% paraformaldehyde and a series of ethanol, respectively. Thereafter, the tissues were embedded in paraffin and cut into sections (5 μm thick) for staining with hematoxylin (H3136, Sigma, USA) and eosin (HE, E4009, Sigma, USA). The histopathological changes in the lung and ileal tissues were observed using a microscope (Eclipse Ci-L, Nikon, Japan).

### 2.10. Terminal deoxynucleotidyl transferase-mediated nick end labeling staining

Terminal deoxynucleotidyl transferase-mediated nick end labeling (TUNEL) assay was employed to assess the apoptosis in the lung tissues. In short, TUNEL staining was performed on the processed lung tissue sections by an *in situ* cell death detection kit (11684795910, Roche, France), as per the supplier's instructions. Finally, the slices were counterstained with 4′,6-diamidino-2-phenylindole for 10 min in the darkness to identify the nucleus. After completing staining, the non-apoptotic cell appeared blue, while the apoptotic cell was green. The slices were viewed using the microscope, and the apoptosis of the lung tissues was exhibited as the amount of TUNEL-positive cells.

### 2.11. Quantitative PCR (qPCR)

The total RNA was isolated from lung tissue homogenate by TRIzol reagents. Then, the total RNA was used to synthesize cDNA using reverse transcription kits (CW2569, CWBIO). Thereafter, quantitative PCR (qPCR) was conducted by SYBR Green qPCR kits (11201ES08, YE SEM, China) and carried out on real-time PCR instruments (LightCycler 96, Roche, Germany). The relative mRNA expressions were calculated as the ratio of GAPDH and analyzed by the 2^−ΔΔCt^ method. The qPCR primer sequences designed in the experiment are presented in Table 1.

**Table 1:** qRT-PCR primers.

**Gene**	**Forward primer**	**Reverse primer**
Mouse IL-1β	TTGAAGTTGACGGACCCCAA	TGTCCTGACCACTGTTGTTTC
Mouse IL-6	GGCGGATCGGATGTTGTGAT	GGACCCCAGACAATCGGTTG
Mouse TNF-α	TCACTGGAGCCTCGAATGTC	TCTGTGAGAAGGCTGTGCA
Mouse iNOS	TTCACGACACCCTTCACCACAA	CCATCCTCCTGCCCACTTCCTC
Mouse Cox-2	CACCCTGACATAGACAGTGAAAG	CTGGGTCACGTTGGATGAGG
Mouse GAPDH	CGAGACACGATGGTGAAGGT	TGCCGTGGGTGGAATCATAC

### 2.12. Immunohistochemical staining

After fixation with paraformaldehyde, the lung and ileal tissues were embedded and cut as usual. Immunohistochemical staining was, then, performed to detect the expressions of TLR4, ZO-1, claudin-1, and occludin. In brief, the sections were deparaffinized and rehydrated prior to quenching in H_2_O_2_ (3%). Upon placing in 5% bovine serum albumin to block non-specific binding, the lung samples were probed with anti-TLR4 antibody (1:100, AF7017, Affinity, China), and the ileal samples were probed with anti-ZO-1 (1:500, ab221547, Abcam, UK), anti-claudin-1 (1:250, ab211737, Abcam, China), and anti-occludin (1:200, ab216327, Abcam, UK). The next day, the slices were washed and then reacted with secondary antibodies, stained with diaminobenzidine and hematoxylin, and finally examined under a microscope.

### 2.13. Western blot analysis

The total protein was isolated from lung and ileal tissue homogenate by Radioimmunoprecipitation assay buffer. Then, bicinchoninic acid kits were applied to detect protein concentration. Afterward, protein extracts were separated by SDS polyacrylamide gel electrophoresis and transferred onto polyvinylidene fluoride membranes. After shaking softly in blocking solution, the membranes were incubated with primary antibodies overnight at 4°C. The following day, the membranes were washed and incubated again with secondary antibodies. After the blots were visualized using enhanced chemiluminescence, data were analyzed by ImageJ software. All information on the primary antibodies is presented in Table 2.

**Table 2:** Antibody information.

**Antibody**	**Source**	**Cat No**.	**Dilutions**
Bax	Affinity	AF0120	1:1,000
Bcl-2	Affinity	AF6139	1:1,000
Caspase-3	CST	9662s	1:1,000
Cleaved-caspase-3	Abcam	ab2302	1:1,000
ZO-1	Proteintech	21773-1-AP	1:1,000
Claudin-1	Affinity	AF0127	1:1,000
Occludin	Affinity	DF7504	1:1,000
TLR4	Affinity	AF7017	1:1,000
Myd88	Affinity	AF5195	1:1,000
p-NF-κB p65	Affinity	AF2006	1:1,000
NF-κB p65	Affinity	AF5006	1:1,000
p-IκBα	Affinity	AF2002	1:1,000
IκBα	Affinity	AF5002	1:1,000
Nrf2	Proteintech	16396-1-AP	1:1,000
HO-1	Proteintech	27282-1-AP	1:1,000
COX-2	Affinity	AF7003	1:1,000
iNOS	Affinity	AF0199	1:1,000
β-actin	Affinity	AF7018	1:10,000
GAPDH	Affinity	AF7021	1:10,000

### 2.14. 16S rRNA gene amplicon sequencing

DNA extraction and 16S rRNA gene sequencing of fecal samples were conducted with the help of BioNovoGene Co., Ltd. (China). In short, the total microbial DNA was extracted from the feces and then quantified by Nanodrop. Agarose gel electrophoresis (1.2%) was utilized to evaluate the quality of extracted DNA. After completing 16S rRNA amplification by PCR, purification, and recovery by magnetic beads, the products were subjected to fluorescence quantitative analysis. Subsequently, the TruSeq Nano DNA LT Library Prep Kit provided by Illumina was employed to construct sequencing libraries.

Raw sequence reads were trimmed and filtered into effective reads, which were further clustered into operational taxonomic units (OTUs) with a similarity >97% by UPARSE. The taxonomy of OTU was carried out by the SILVA database. In addition, the α-diversity of microbiota was analyzed by Shannon and Simpson indices, and the β-diversity of microbiota was assessed by weighted UniFrac distance and visualized with non-metric multidimensional scaling (NMDS) plot and analysis of similarities (ANOSIM) test. Additionally, linear discriminant analysis effect size was applied to taxonomic discovery analysis for microbial biomarker discovery.

### 2.15. Statistical analysis

The data were analyzed by SPSS 19.0 and presented as mean ± SD. Differences between multiple groups were compared by analysis of variance (ANOVA) and Tukey's tests. The Kruskal–Wallis *H*-test was applied when variances were not homogeneous. *p* < 0.05 was considered statistically significant. R software was used to perform amplicon sequencing statistical analysis. Furthermore, the Wilcoxon test was utilized to calculate the microbial α-diversity index, while the ANOSIM non-parametric test was conducted to compare microbial β-diversity between the two groups.

## 3. Results

### 3.1. FMT from HUC-MSC-treated mice counteracted the upregulation of BALF inflammatory cell counts and alleviated lung injury in ALI mice

**Figure 2:**
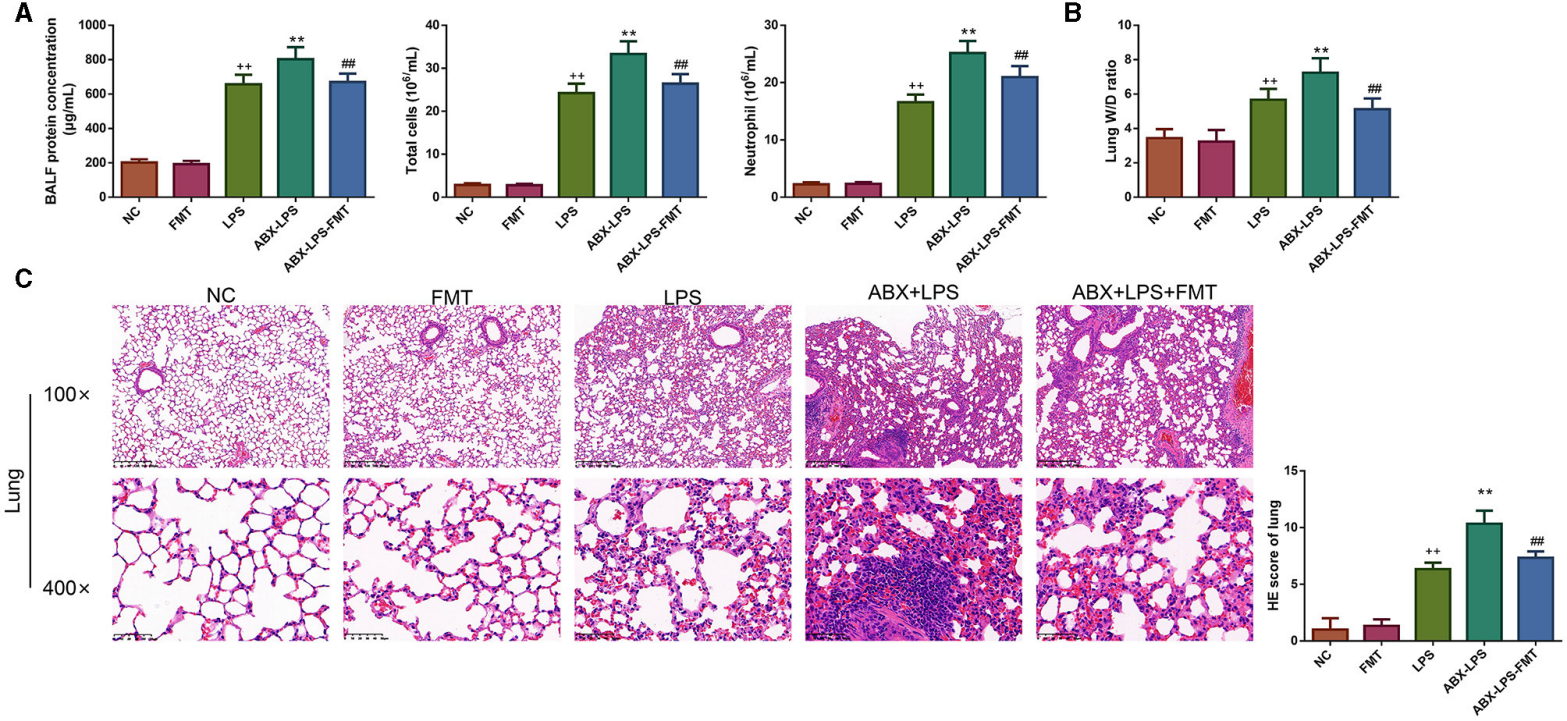
FMT from HUC-MSC-treated mice decreased BALF inflammatory cell counts and attenuated lung injury in ALI mice. Following FMT treatment, the protein concentration, total cells, and neutrophils were detected in the BALF **(A)**, and the W/D ratio in the lung was measured **(B)**, moreover, the pathological changes of the lung tissues were observed by HE staining **(C)**. The magnification for HE staining was 100× and 400×. BALF analysis and HE staining for the lung tissues were repeated three times, and the W/D ratio in the lung was detected once for every mouse. ^++^*p* < 0.01 vs. NC, **p < 0.01 vs. LPS, ^##^*p* < 0.01 vs. ABX + LPS. The results were presented as mean ± SD. HUC-MSCs, human umbilical cord mesenchymal stromal cells; BALF, bronchoalveolar lavage fluid; ALI, acute lung injury; W/D, wet to dry; HE, hematoxylin and eosin.

As shown in Figure 2A, LPS treatment leads to an obvious upregulation of the concentrations of total protein, the counts of total cells, and neutrophils in BALF of ALI rats (*p* < 0.01). Consistent with this, after mice were exposed to LPS, the lung W/D ratio increased clearly (*p* < 0.01), and the lung exhibited serious injury (Figures 2B, C); specifically, the remarkable thickening of the alveolar septum and wall was observed. In addition, from the results of HE staining, we also observed pulmonary capillary edema and congestion in LPS mice. When LPS mice were pre-treated with ABX, all the phenomena described were more pronounced (*p* < 0.01). However, FMT treatment effectively reversed the increase of total protein, total cells, and neutrophils in BALF, reduced the W/D ratio in the lung, and improved lung injury induced by LPS and ABX; the alveolar septa were thinning, and inflammatory cell infiltration was alleviated (*p* < 0.01).

### 3.2. FMT from HUC-MSC-treated mice restored immune cell number in the peripheral blood of ALI mice

**Figure 3:**
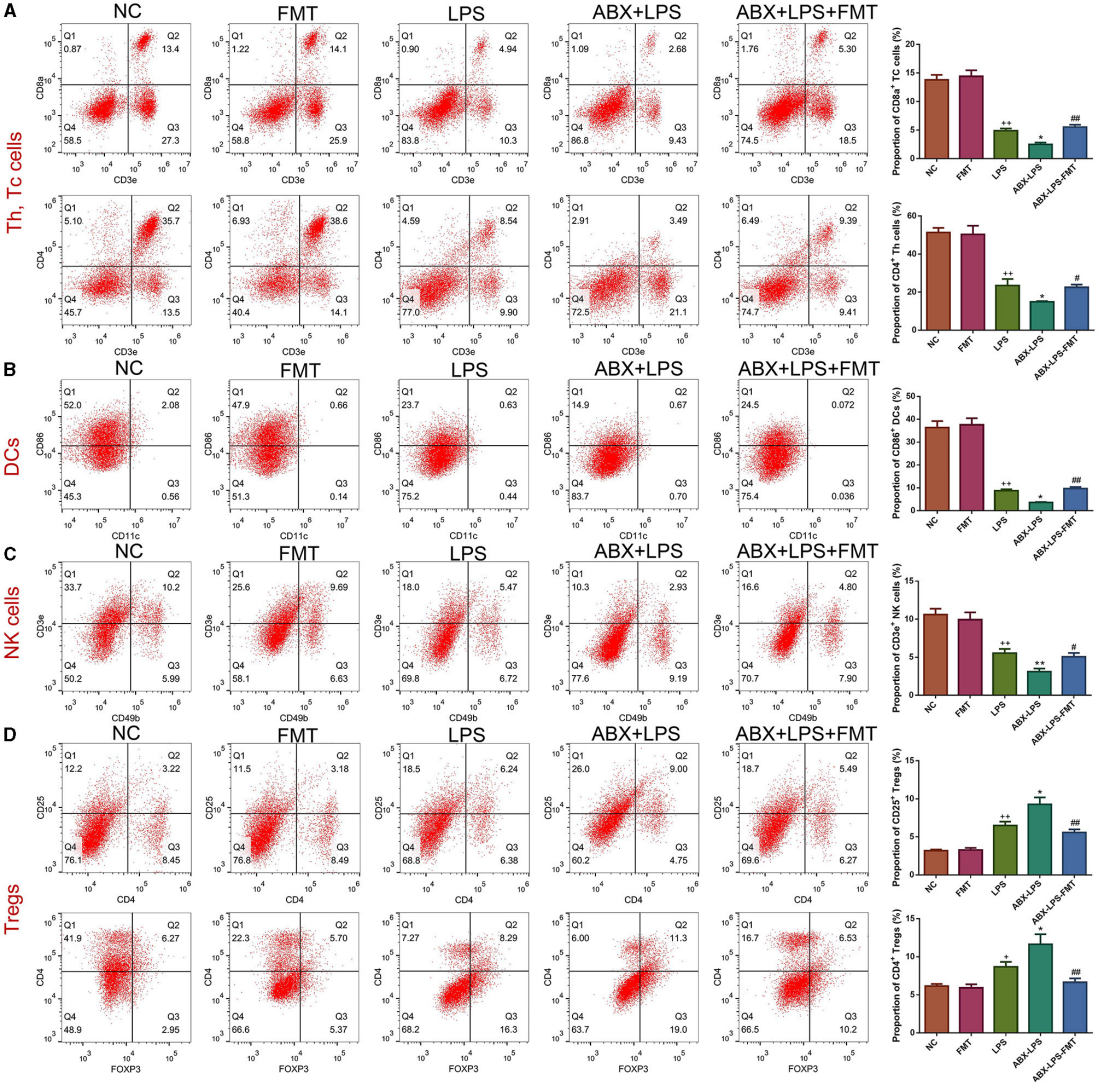
FMT from HUC-MSC-treated mice restored the immune system of the peripheral blood in ALI mice. After treatment with FMT, Th and Tc cells **(A)**, DCs **(B)**, NK cells **(C)**, and Tregs **(D)** in the peripheral blood were evaluated by flow cytometry. Peripheral blood immune cell analysis was repeated three times. ^+^*p* < 0.05 and ^++^*p* < 0.01 vs. NC, *p < 0.05 and **p < 0.01 vs. LPS, ^#^*p* < 0.05 and ^##^*p* < 0.01 vs. ABX + LPS. The results were presented as mean ± SD.

Given that innate immune cells are crucial in ALI development, the percentage of immune cells was detected in the study. Flow cytometry results revealed that LPS exposure caused a significant increase in the percentages of CD4^+^ Th, CD8a^+^ Tc, CD86^+^ DC, and CD3e^+^ NK cells but blocked the percentages of CD25^+^ Tregs and CD4^+^ Tregs cells (*p* < 0.05, Figure 3). Interestingly, ABX pre-treatment worsened this situation (*p* < 0.05). As expected, it can be observed from Figure 3 that FMT treatment moderately attenuated ABX- and LPS-stimulated immune cell changes (*p* < 0.05).

### 3.3. FMT from HUC-MSC-treated mice blunted the apoptosis of the lung tissue in ALI mice

**Figure 4:**
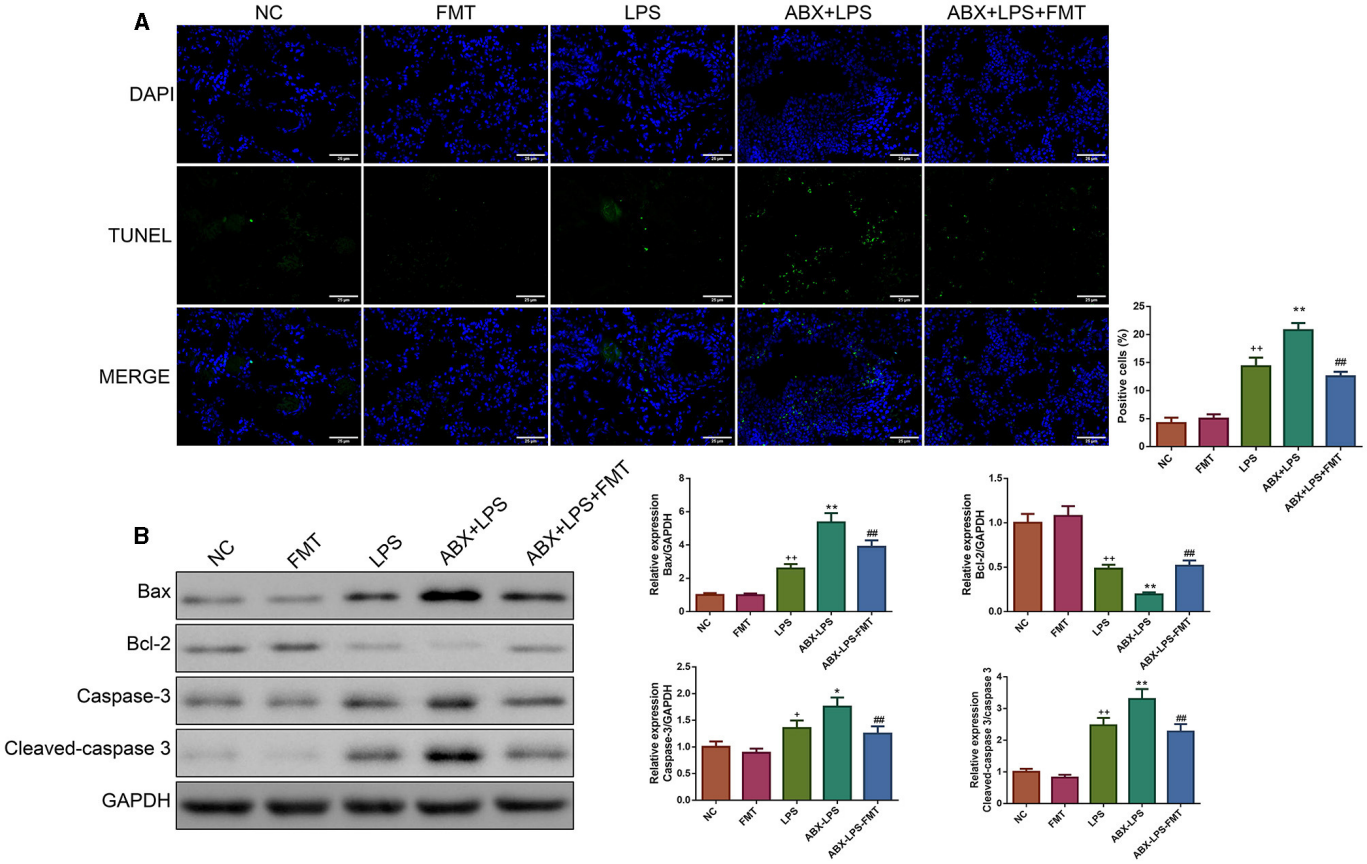
FMT from HUC-MSC-treated mice reduced the apoptosis of the lung tissues in ALI mice. After FMT, the apoptosis of the lung tissues was evaluated by TUNEL staining with the magnification of ×400 **(A)**, and the expressions of apoptosis-associated protein (including Bax, Bcl-2, caspase-3, and cleaved-caspase-3) in the lung tissues were tested by Western blot **(B)**. TUNEL and Western blot analyses for the lung tissues were repeated three times. ^+^*p* < 0.05 and ^++^*p* < 0.01 vs. NC, *p < 0.05 and **p < 0.01 vs. LPS, ^##^*p* < 0.01 vs. ABX + LPS. The results were presented as mean ± SD. TUNEL, Terminal Deoxynucleotidyl Transferase-Mediated Nick End Labeling.

The percentage of apoptotic cells was raised remarkably in the lung tissues of LPS-exposed mice (*p* < 0.01, Figure 4A). Accordingly, ABX pre-treatment deteriorated the apoptosis of the lung tissues in LPS mice (*p* < 0.01). Nevertheless, FMT treatment effectively counteracted the upregulation of apoptosis in the lung tissues after ABX and LPS stimulation (*p* < 0.01). The apoptosis-related proteins in the lung tissues were utilized to verify the extent of apoptosis. Unanimously, we found that LPS and ABX increase the Bax, caspase-3, and cleaved-caspase-3 protein expressions and decrease the Bcl-2 protein expression (*p* < 0.05, Figure 4B); however, those changes were offset by the FMT treatment (*p* < 0.01).

### 3.4. FMT from HUC-MSC-treated mice prevented oxidative stress and inflammation response in ALI mice

**Figure 5:**
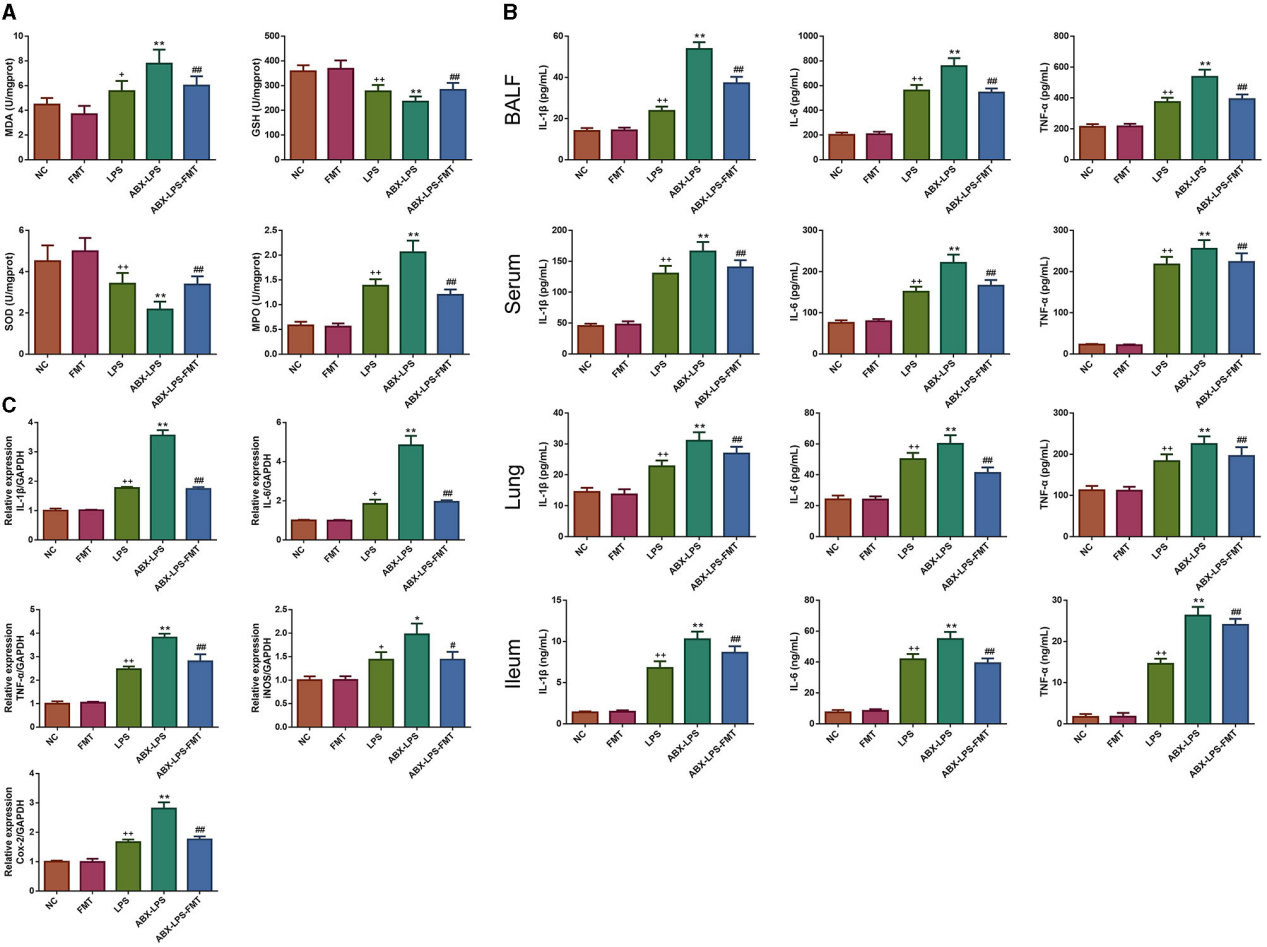
FMT from HUC-MSC-treated mice prevented oxidative stress and inflammation in ALI mice. Upon FMT treatment, the levels of MDA, GSH, SOD, and MPO were measured in the lung tissues **(A)**. In addition, the contents of IL-1β, IL-6, and TNF-a in the BALF, serum, lung, and ileal tissues were assessed by ELISA **(B)**, the expressions of IL-1β, IL-6, TNF-a, iNOS, and Cox-2 mRNAs in the lung tissues were detected using qPCR **(C)**. MDA, GSH, SOD, and MPO levels in the lung tissues and ELISA assay for the BALF, serum, and lung were measured once for every mouse; in addition, qPCR analysis for the lung tissues was repeated three times. ^+^*p* < 0.05 and ^++^*p* < 0.01 vs. NC, *p < 0.05 and **p < 0.01 vs. LPS, ^#^*p* < 0.05 and ^##^*p* < 0.01 vs. ABX + LPS. The results were presented as mean ± SD. ELISA, enzyme-linked immunosorbent assay; qPCR, quantitative PCR.

Next, the effect of FMT from HUC-MSC-treated mice on the inhibition of oxidative stress and inflammation response was determined. As shown in Figure 5A, the lung tissues of LPS-stimulated mice had higher MDA and MPO levels and lower GSH and SOD levels than normal control mice (*p* < 0.05). In addition, ELISA results also revealed that the LPS challenge led to serious inflammation in the BALF, serum, lung, and ileal tissues (Figure 5B). ABX pre-treatment further worsened those changes. However, ABX- and LPS-induced increases of MDA, MPO, IL-1β, IL-6, and TNF-α and reduction of GSH and SOD were effectively restored by FMT treatment (*p* < 0.01). Then, qPCR was conducted to further assess the antioxidant and anti-inflammatory effects of FMT treatment. It can be observed from Figure 5C that IL-1β, IL-6, TNF-α, iNOS, and Cox-2 mRNA expressions were significantly raised in the LPS and ABX-LPS groups (*p* < 0.05), but upon FMT treatment, the upregulation of these mRNA was effectively relieved (*p* < 0.05).

### 3.5. FMT from HUC-MSC-treated mice improved the barrier function and inhibited the apoptosis of ileal tissues in ALI mice

**Figure 6:**
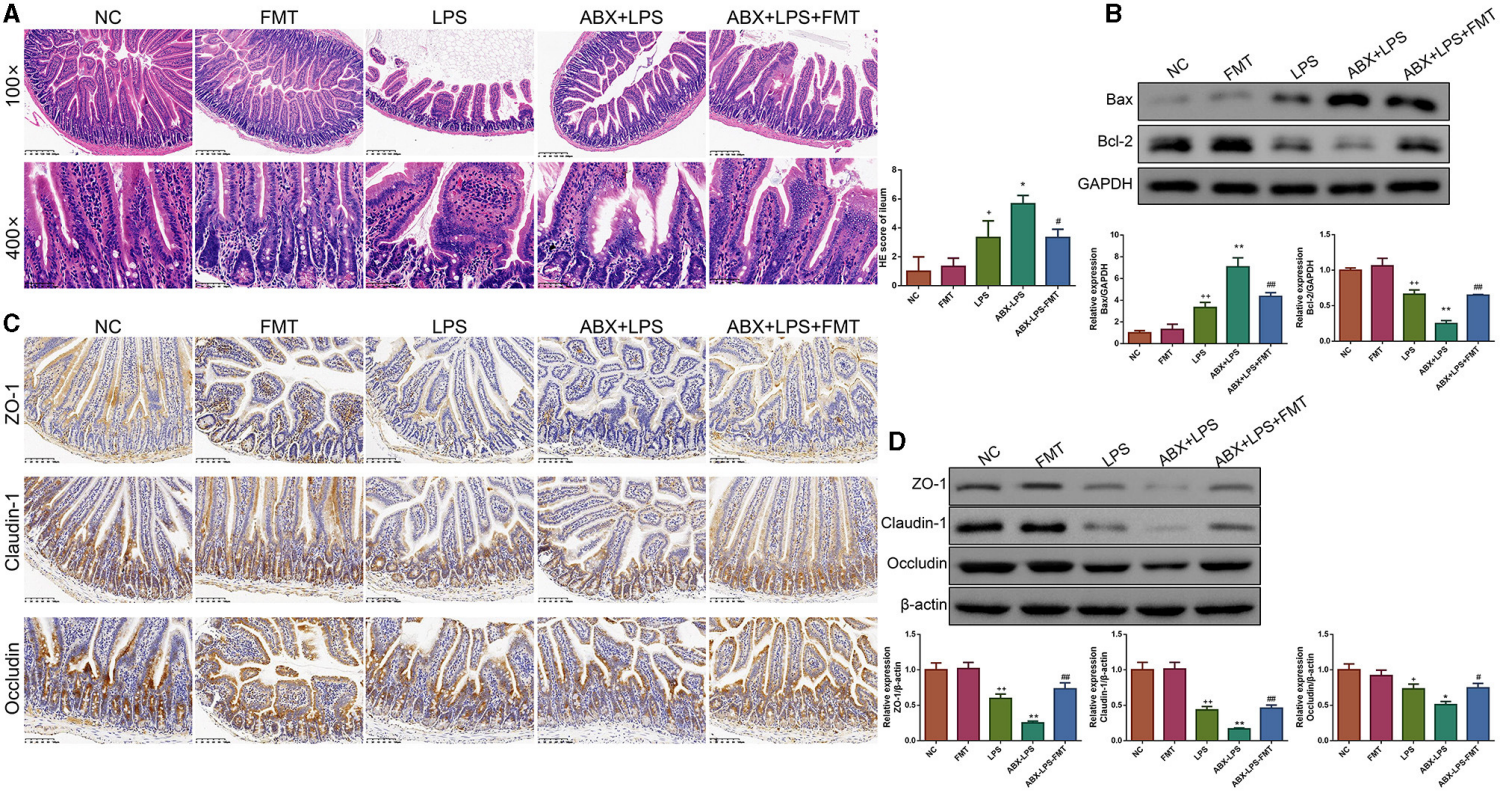
FMT from HUC-MSC-treated mice restored barrier integrity of the ileum tissues in ALI mice. After treatment with FMT, the pathological changes in the ileum tissues were analyzed by HE **(A)**, and the protein expressions of Bax and Bcl-2 in the ileum tissues were measured by Western blot **(B)**, moreover, immunohistochemical staining **(C)** and Western blot **(D)** were employed to measure ZO-1, claudin-1, and occludin protein expressions. The magnification for immunohistochemical staining was 200×, and for HE staining was 100× and 400×. HE staining, Western blot, and immunohistochemical staining for the ileum tissues were repeated three times. ^+^*p* < 0.05 and ^++^*p* < 0.01 vs. NC, *p < 0.05 and **p < 0.01 vs. LPS, ^#^*p* < 0.05 and ^##^*p* < 0.01 vs. ABX + LPS. The results were presented as mean ± SD.

It is well-known that injured ileal tissues and impaired barrier function contribute to the development of ALI (Xu Y. et al., 2021). Thus, we checked whether FMT from HUC-MSC-treated mice could alleviate the injured degree, inhibit apoptosis, and improve the impaired barrier function for the ileal tissues. It can be observed that after stimulation by LPS, the ileal tissues appeared with obvious injury (Figure 6A). Accordingly, the situation was more serious in the ABX-LPS group. As expected, FMT treatment attenuated ABX- and LPS-induced ileal tissue injury. In addition, following LPS stimulation, Bax protein expression was increased, while Bcl-2 protein expression was decreased (*p* < 0.01), the situation further deteriorated in the ABX + LPS group (*p* < 0.01, Figure 6B). However, FMT from HUC-MSC-treated mice decreased Bax protein expression but increased Bcl-2 protein expression (*p* < 0.01). The expressions of ZO-1, claudin-1, and occludin are crucial for maintaining barrier function (Shi et al., 2020). The results of immunohistochemical staining revealed that ZO-1, claudin-1, and occludin protein expressions were obviously decreased upon ABX and LPS stimulation (*p* < 0.05, Figure 6C). Of note, FMT treatment elevated the decrease in ZO-1, claudin-1, and occludin protein expressions (*p* < 0.05). As expected, the Western blot results exhibited a similar trend as the results of the immunohistochemical staining (Figure 6D).

### 3.6. FMT from HUC-MSC-treated mice restored the TLR4/NF-κb and Nrf2/HO-1 pathways in ALI mice

**Figure 7:**
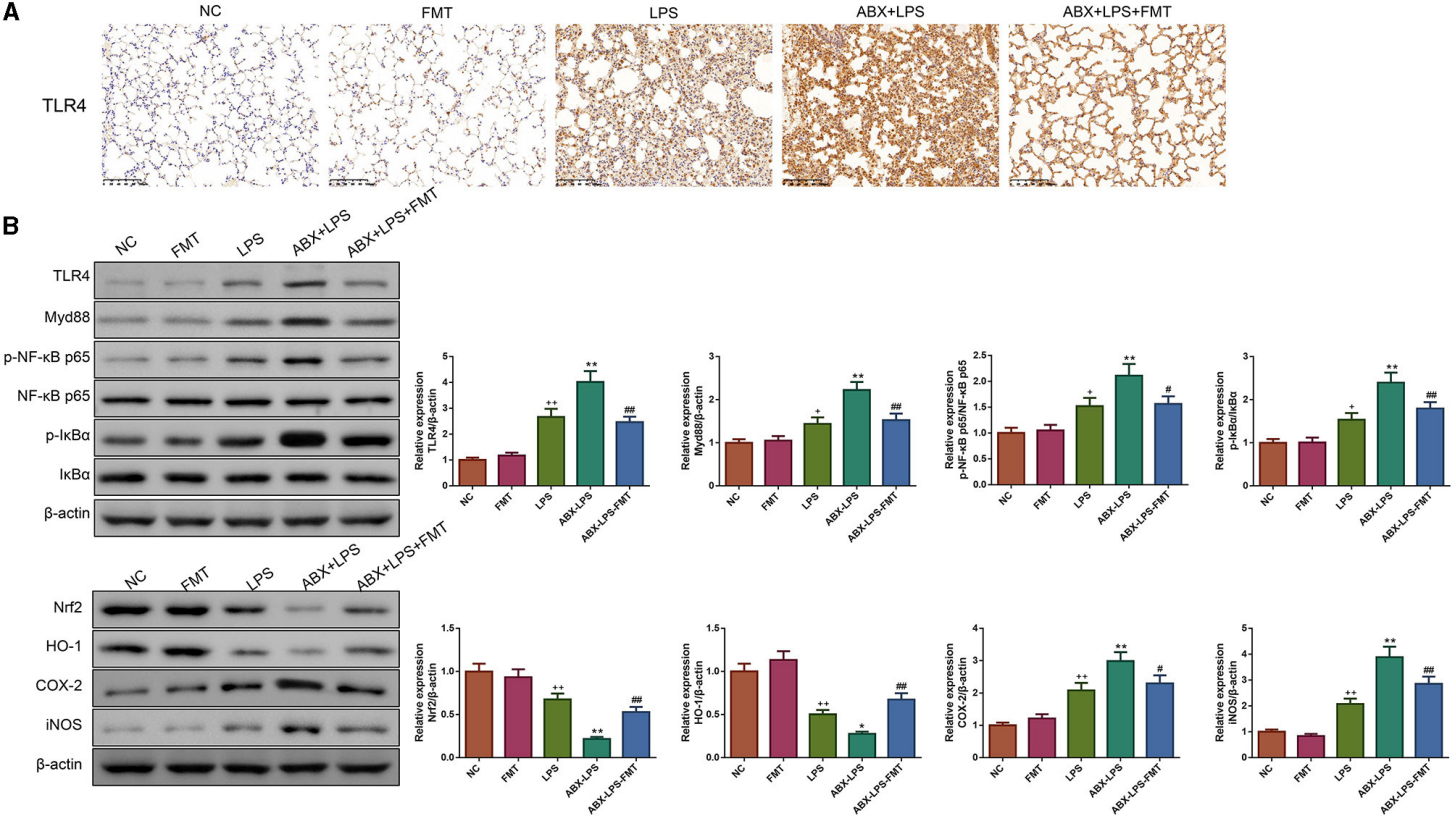
FMT from HUC-MSC-treated mice recover the TLR4/NF-κB and Nrf2/HO-1 pathways for the lung tissues of ALI mice. Immunohistochemical staining was conducted to detect the expression of TLR4 in the lung tissues with the magnification of ×200 **(A)**, and Western blot was performed to detect the protein expressions of TLR4, Myd88, Nrf2, HO-1, COX-2, and iNOS and the phosphorylation of NF-κB and IκBα in the lung tissues **(B)**. Immunohistochemical staining and Western blot for the lung tissues were repeated three times. ^+^*p* < 0.05 and ^++^*p* < 0.01 vs. NC, *p < 0.05 and **p < 0.01 vs. LPS, ^#^*p* < 0.05 and ^##^*p* < 0.01 vs. ABX + LPS. The results were presented as mean ± SD.

**Figure 8:**
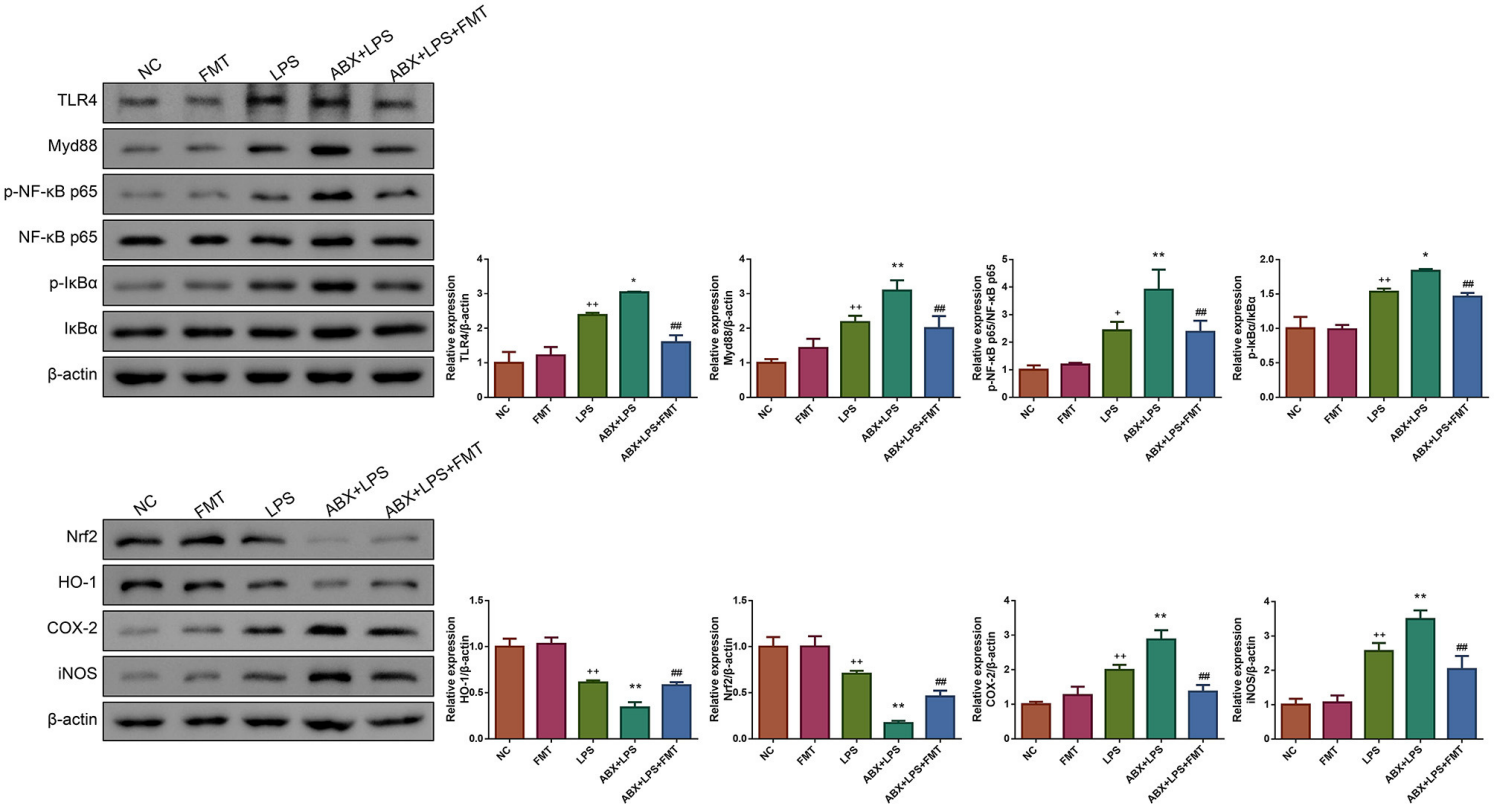
FMT from HUC-MSC-treated mice recovers the TLR4/NF-κB and Nrf2/HO-1 pathways for the ileum tissues of ALI mice. Western blot was performed to detect the protein expressions of TLR4, Myd88, Nrf2, HO-1, COX-2, and iNOS and the phosphorylation of NF-κB and IκBα in the ileum tissues. Western blot for the ileum tissues was repeated three times. ^+^*p* < 0.05 and ^++^*p* < 0.01 vs. NC, *p < 0.05 and **p < 0.01 vs. LPS, ^##^*p* < 0.01 vs. ABX + LPS. The results were presented as mean ± SD.

**Figure 9:**
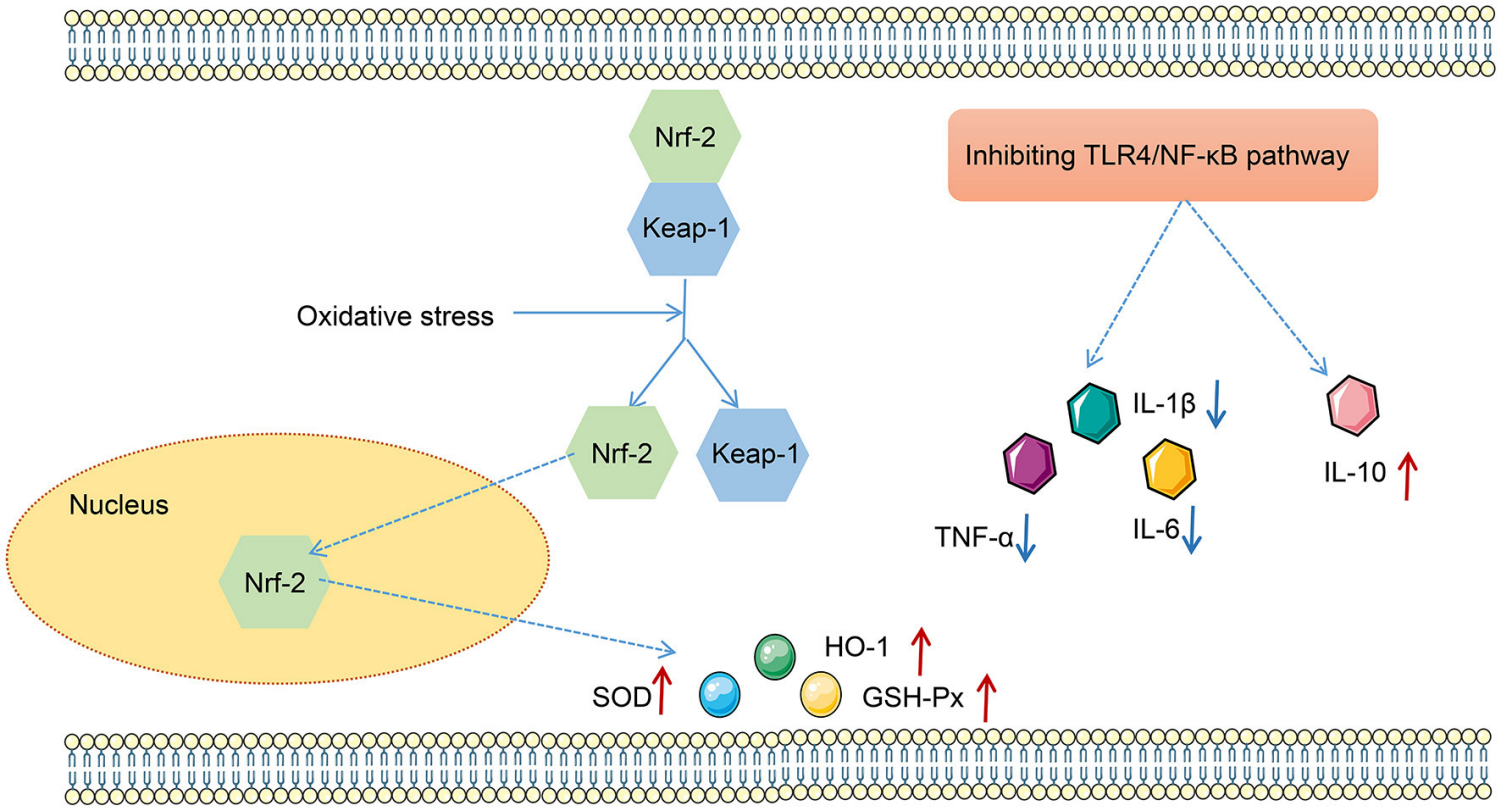
The mechanism map of effects of the TLR4/NF-κB and Nrf2/HO-1 pathways on oxidative stress and inflammation.

LPS has been found to play pro-oxidative and pro-inflammatory roles by the TLR4/NF-κB and Nrf2/HO-1 pathways (Xin et al., 2020). Therefore, the impact of FMT from HUC-MSC-treated mice on the TLR4/NF-κB and Nrf2/HO-1 pathways was tested. Immunohistochemical staining and Western blot analysis demonstrated that ABX and LPS treatments raised the protein expressions of TLR4, Myd88, COX-2, and iNOS, as well as the phosphorylation of NF-κB and IκBα in the lung tissues of mice, but decreased the protein expressions of Nrf2 and HO-1 (*p* < 0.05, Figure 7). Notably, FMT treatment restored the expressions and phosphorylation of TLR4/NF-κB and Nrf2/HO-1 pathway-related proteins in the lung tissues from ABX- and LPS-treated mice (*p* < 0.05). The results obtained from the ileal tissues were similar to those from the lung tissues (Figure 8). The mechanistic diagram is presented in Figure 9.

### 3.7. FMT from HUC-MSC-treated mice alleviated the dysbiosis of GM in ALI mice

**Figure 10:**
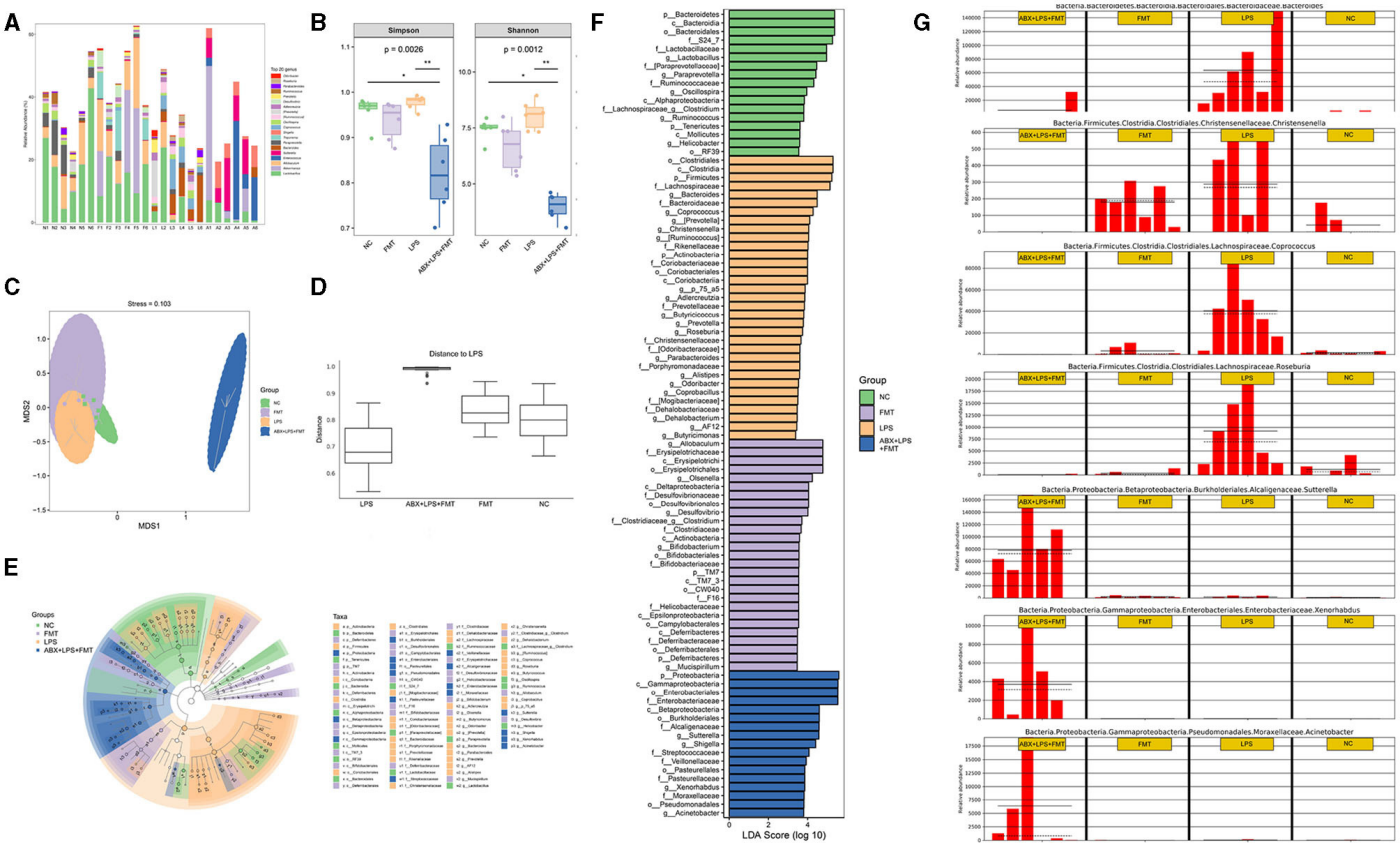
FMT from HUC-MSC-treated mice alleviated the disorder of GM in ALI mice. After FMT treatment, the top 20 bacterial genera with the highest relative abundance among the groups were tested **(A)**. The microbial α-diversity was analyzed by Shannon and Simpson indices **(B)**. The microbial β-diversity was analyzed by NMDS **(C)** and ANOSIM **(D)**. LEfSe was carried out to discriminate the different taxa from the fecal samples of the mice **(E, F)**. **(G)** The relative abundance of *Bacteroides, Christensenella, Coprococcus, Roseburia, Xenorhabdus, Sutterella*, and *Acinetobacter* was compared between the groups. In total, 24 fecal samples from 4 groups were applied for 16S rRNA gene amplicon sequencing. *p < 0.05 and **p < 0.01. GM, gut microbiota; NMDS, non-metric multidimensional scaling; ANOSIM, analysis of similarities; LEfSe, linear discriminant analysis effect size.

Further sequencing of the 16S rRNA gene was applied to detect the microbial profiles of the fecal samples from mice in the NC, FMT, LPS, and ABX-LPS + FMT groups. The top 20 bacterial genera are presented in Figure 10A in terms of the relative abundance. In addition, microbial α-diversity analyzed by Shannon and Simpson indices revealed that the community diversity of microbe in the LPS group was higher than those of the NC and FMT groups, despite the difference was not significant (Figure 10B). However, the community diversity of microbe from ABX-LPS + FMT mice was effectively decreased (*p* < 0.01). Additionally, microbial β-diversity was also analyzed by NMDS and ANOSIM, and the results revealed that the microbial community structure of ABX-LPS + FMT mice changed obviously (Figures 10C, D). Then, we further screen the potential GM change by FMT treatment in LPS mice. LEfSe analysis was conducted to detect the bacterial taxa that differed significantly among the groups (Figures 10E, F). Specifically, at the genus level, we found that relative to the NC group, the relative abundances of *Bacteroides, Christensenella, Coprococcus*, and *Roseburia* were significantly elevated in the LPS group. However, the relative abundances of *Bacteroides, Christensenella, Coprococcus*, and *Roseburia* were decreased obviously in the ABX-LPS + FMT group. Furthermore, the relative abundances of *Xenorhabdus, Sutterella*, and *Acinetobacter* obviously increased in the ABX-LPS + FMT group (Figure 10G).

## 4. Discussion

Despite immense efforts have been made and substantial achievements have been achieved to develop new therapies for ALI, effective treatments for ALI continue to be limited (Wang et al., 2021). Recently, scholars have demonstrated that MSCs have excellent immunomodulatory functions in numerous lung disorders, including ALI (Peng W. et al., 2021). A published study has revealed that MSCs derived from the bone marrow, chorion, and lung can manage LPS-induced ALI by suppressing inflammatory responses and increasing the Tregs/Th17 cell ratio (Wang L. et al., 2022). In addition, our previous study has also proven that HUC-MSCs can be used to treat ALI effectively by redefining gut microbiota. At the same time, researchers have demonstrated the composition, structure, and distribution of the intestinal flora were remarkably different between the ALI and normal mice (Li et al., 2014). However, elevating short-chain fatty acid generation and remodeling intestinal flora disorder can alleviate LPS effectively (Peng L.-Y. et al., 2021). Given that FMT is an effective method to improve the altered GM diversity and exhibited more cost-effectiveness than antimicrobial treatment in some cases (Hu et al., 2019), we hypothesized that FMT from HUC-MSC-treated mice may have the potential to treat ALI.

Using appropriate animal models is crucial for studying the effects and mechanisms of drugs for diseases. Currently, LPS-induced ALI has become the most common and reliable animal model to test the potential therapies for ALI (de Souza Xavier Costa et al., 2017). It is well-known that intratracheal injection of LPS will induce the generation of inflammatory factors and stimulate the neutrophils to migrate to the lung, thereby inducing systemic inflammation and lung damage (Li et al., 2021). In this study, the BALF protein concentration, total cell number, neutrophil number, and lung W/D ratio increased obviously after LPS administration, which implied that lung damage happened in mice. The pathological injuries of the lung further demonstrated it. Nevertheless, all the changes induced by LPS were restored by FMT treatment, which indicated that FMT from HUC-MSC-treated mice can ameliorate LPS-stimulated ALI.

A body of evidence has revealed that inflammation is the key mechanism for ALI pathogenesis (Chen et al., 2022). Specifically, inflammatory responses can increase alveolar capillary permeability, which facilitates the leak of fluid, thereby promoting the formation of lung edema (Zhao and Bie, 2020). Additionally, inflammation-induced oxidative stress also plays a key function in the development of ALI (Du et al., 2021). Under a healthy condition, the scavenging and generation of reactive oxygen species (ROS) are balanced in the body; however, inflammation will disrupt this balance and stimulate the production of ROS (Lv et al., 2016). Excessive ROS will attack polyunsaturated fatty acids, generating lipid peroxides (such as MDA) and leading to the injury of tissues (Huang et al., 2016). On the other hand, excessive ROS also contributes to the release of inflammatory factors in turn, thus entering a vicious cycle (Wang et al., 2019). This study found that inflammation and oxidative stress were aggravated in response to LPS treatment, which aligned with the symptoms observed in ALI patients (Gong et al., 2023). However, FMT treatment could effectively inhibit the inflammatory response and oxidative stress stimulated by LPS, suggesting that FMT from HUC-MSC-treated mice may alleviate ALI by repressing inflammatory response.

TLR4 is thought to be the specific receptor for LPS in mammals and is essential in the development of inflammatory response, while the NF-κB pathway plays a central role in the modulation of inflammatory response (Yang W. et al., 2022). Activation of the NF-κB pathway promotes the excessive release of pro-inflammatory factors (TNF-α, IL-6, IL-1β, and so on) but inhibits the secretion of anti-inflammatory factors (interleukin 10); the imbalance between pro-inflammatory and anti-inflammatory factors will result in ALI (Han et al., 2019). Previous research by Li et al. (2016) has reported that MSCs obtained from the bone marrow have the potential to treat ALI by suppressing inflammation by downregulating the TLR2 and 4/NF-κB pathways. In addition, when the body suffers from inflammation and oxidative stress, Nrf2, a basic leucine zipper protein, will modulate the expression of proteins that participate in protection from oxidative injuries (Lei et al., 2020). In normal physiological conditions, Nrf2 exists in the cytoplasm by binding with Keap-1 (Zhang Z. et al., 2022). However, when subjected to oxidative stress, Nrf2 will dissociate from Keap-1, combined with ARE, and subsequently transported to the nucleus to induce the expressions of target genes (such as HO-1, GSH-Px, and SOD), thereby protecting cells from oxidative stress (Liberti et al., 2020). Drugs that activate the Nrf2/HO-1 pathway have been studied for treating diseases caused by oxidative stress (Xu C. et al., 2021). It has already been demonstrated that MSCs can activate HO-1 and Nrf2 and suppress inflammation, cell apoptosis, and oxidative stress in the lung tissues, thereby improving ALI (Zhang et al., 2019b). In addition, another study has revealed that pre-treating HUC-MSCs with pyrogallol improves LPS-stimulated lung injury and inflammation by activating the Nrf2/HO-1 pathway (Zhang Y. et al., 2022). Similarly, this research found that FMT from HUC-MSC-treated mice inhibited the TLR4/NF-κB pathway and activated the Nrf2/HO-1 pathway, which might explain the improving mechanism of FMT on ALI.

The intestinal barrier is pivotal in sustaining intestinal balance, and impairment of its function may result in the displacement of harmful intestinal substances to the circulatory system (Wang R. et al., 2022). The expressions of ZO-1, claudins, and occludin in the ileum are essential in modulating intestinal permeability (Li L. et al., 2023). In addition, it is widely accepted by researchers that injured ileal tissues and impaired barrier function are involved in ALI development (Xu Y. et al., 2021). In the study, we found that FMT from HUC-MSC-treated mice could improve the impaired degree of the ileum and upregulated the expressions of ZO-1, claudins, and occludin proteins in the ileum, suggesting that FMT from HUC-MSC-treated mice can improve intestinal barrier function for ALI mice.

The composition of GM can also influence the development of ALI (Kosyreva et al., 2020). By alerting the composition and metabolisms of GM and modulating the balance between beneficial and harmful bacteria, the injured lung can be alleviated and lung immunity can be enhanced (Liu et al., 2020a). *Bacteroides* is a microbe recognized as a potential mucus degrader; research has reported that pulmonary *Bacteroides* are negatively associated with lung function (Yang Y.-S. H. et al., 2022). Additionally, *Christensenella* dominates the lungs of patients with idiopathic pulmonary fibrosis or lung cancer, while the *Roseburia* genus was elevated in the fecal sample of non-small cell lung cancer patients (D'Alessandro-Gabazza et al., 2018). Moreover, inhibiting the relative abundance of *Coprococcus* in the gut can improve immune function and lung function (Jia et al., 2022). It is well-known that *Xenorhabdus* can produce various antimicrobial compounds (Dreyer et al., 2019), and the abundance of *Sutterella* is negatively correlated with ileal-pouch inflammation (Olaisen et al., 2021). In the present study, *Bacteroides, Christensenella, Roseburia*, and *Coprococcus* were reduced, while *Xenorhabdus, Sutterella*, and *Acinetobacter* were increased after FMT treatment, which confirmed that FMT from HUC-MSC-treated mice alleviated LPS-induced ALI by regulating the relative abundance of specific GM. In future, we will gavage *Xenorhabdus, Sutterella*, and *Acinetobacter* to ALI mice, so as to further investigate whether these GM can ameliorate ALI and explore the specific mechanism.

However, the main limitation of the study is the absence of a control group, i.e., letting the mice in the ABX + LPS group receive FMT from healthy mice. In future, we will treat ABX + LPS mice with FMT from healthy mice to make the key funding of the study more convincing.

In summary, this study demonstrated that FMT from HUC-MSC-treated mice may improve ALI by inhibiting inflammation and modulating GM abundance (Figure 9). Additionally, the TLR4/NF-KB and Nrf2/HO-1 pathways may also take part in the improvement of FMT on ALI, which needed further exploration by using corresponding agonist and inhibitor. These discoveries provided powerful evidence for the protective function of FMT from HUC-MSC-treated mice on ALI.

## Data availability statement

The data presented in the study are deposited in the National Center for Biotechnology Information (NCBI) BioProject database, accession number PRJNA1016527.

## Ethics statement

All animal related experiments were carried out with the evaluation and supervision of the Animal Experimentation Ethics Committee of Zhejiang Eyong Pharmaceutical Research and Development Center [certificate No. SYXK (Zhe) 2021-0033] and in compliance with the guidelines of the Institutional Animal Care and Use Committee.

## Author contributions

FH, EC, and BW: conceptualization and writing—reviewing and editing. EC, LLv, BW, LLi, HL, NC, and WC: data curation. EC and LLv: writing—original draft preparation. EC and BW: funding acquisition. All authors have read and agreed to the published version of the manuscript.

## Supplementary material

Supplementary Figure 1 The microflora homeostasis in feces of mice treated with or without HUC-MSCs. After HUC-MSC treatment, the top 20 bacterial families with the highest relative abundance among the groups were tested **(A)**. The microbial α-diversity **(B)** and β-diversity **(C)** were statistically analyzed in the feces of different groups. The heatmap was applied to analyze the differences in the genus abundance of different microflora in feces **(D)**. *n* = 6. Note: HUC-MSCs, human umbilical cord mesenchymal stromal cells.
