# Supplementary figures and images for: Fecal microbiota transplantation from HUC-MSC-treated mice alleviates acute lung injury in mice through anti-inflammation and gut microbiota modulation
Source: Front Microbiol. 2023 Sep 28;14:1243102. doi: 10.3389/fmicb.2023.1243102 (PMC10569429; doi:10.3389/fmicb.2023.1243102)

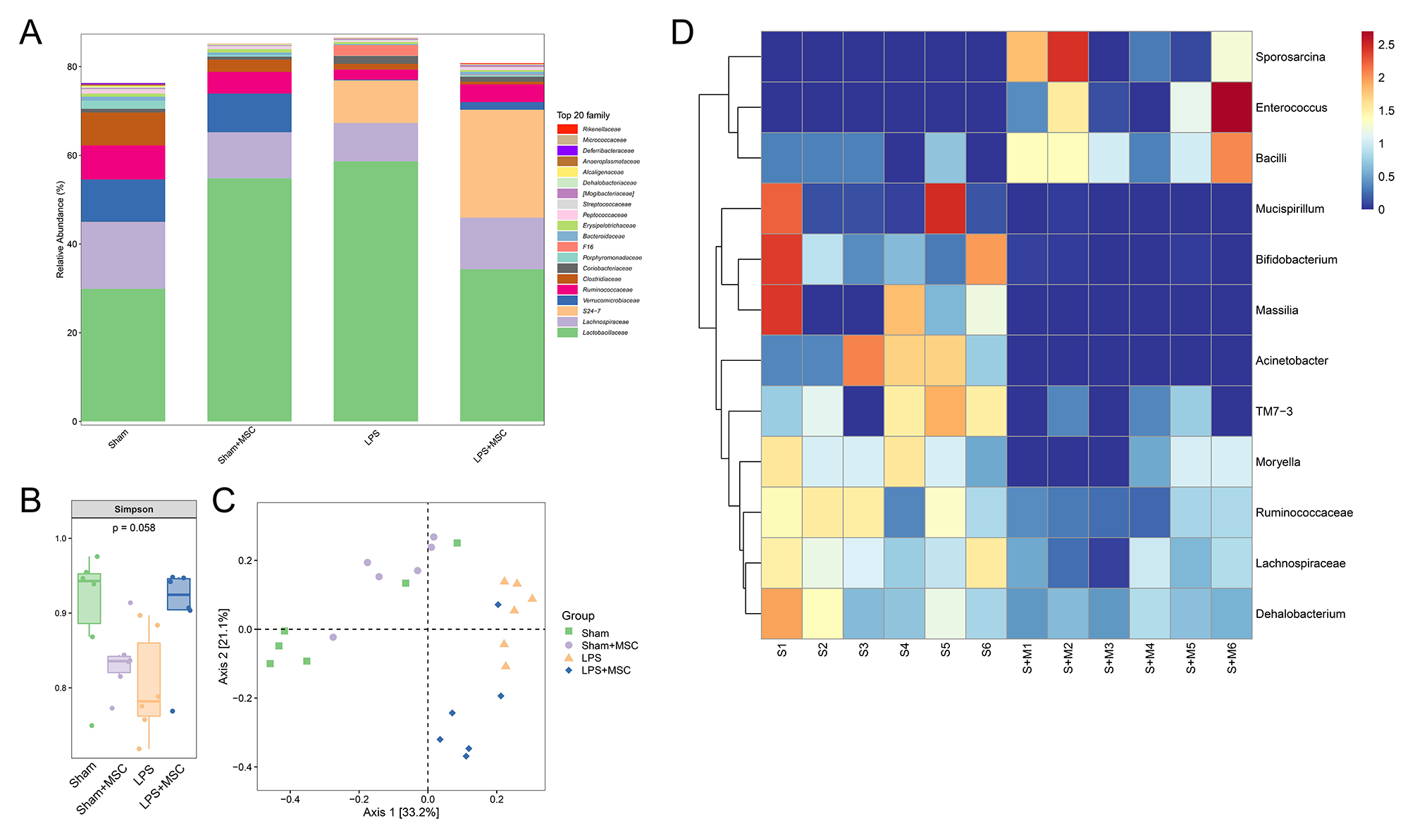

Supplement: Supplementary Figure 1 — The microflora homeostasis in feces of mice treated with or without HUC-MSCs. After HUC-MSC treatment, the top 20 bacterial families with the highest relative abundance among the groups were tested (A). The microbial α-diversity (B) and β-diversity (C) were statistically analyzed in the feces of different groups. The heatmap was applied to analyze the differences in the genus abundance of different microflora in feces (D). n = 6. Note: HUC-MSCs, human umbilical cord mesenchymal stromal cells. [file Image_1.tif]
